# Parishin from* Gastrodia elata* Extends the Lifespan of Yeast via Regulation of Sir2/Uth1/TOR Signaling Pathway

**DOI:** 10.1155/2016/4074690

**Published:** 2016-06-27

**Authors:** Yanfei Lin, Yujuan Sun, Yufang Weng, Akira Matsuura, Lan Xiang, Jianhua Qi

**Affiliations:** ^1^College of Pharmaceutical Sciences, Zhejiang University, 866 Yu Hang Tang Road, Hangzhou, China; ^2^Department of Nanobiology, Graduate School of Advanced Integration Science, Chiba University, Chiba 263-8522, Japan

## Abstract

Parishin is a phenolic glucoside isolated from* Gastrodia elata*, which is an important traditional Chinese medicine; this glucoside significantly extended the replicative lifespan of K6001 yeast at 3, 10, and 30 *μ*M. To clarify its mechanism of action, assessment of oxidative stress resistance, superoxide dismutase (SOD) activity, malondialdehyde (MDA), and reactive oxygen species (ROS) assays, replicative lifespans of* sod1*,* sod2*,* uth1*, and* skn7* yeast mutants, and real-time quantitative PCR (RT-PCR) analysis were conducted. The significant increase of cell survival rate in oxidative stress condition was observed in parishin-treated groups. Silent information regulator 2 (*Sir2*) gene expression and SOD activity were significantly increased after treating parishin in normal condition. Meanwhile, the levels of ROS and MDA in yeast were significantly decreased. The replicative lifespans of* sod1*,* sod2*,* uth1,* and* skn7* mutants of K6001 yeast were not affected by parishin. We also found that parishin could decrease the gene expression of* TORC1*, ribosomal protein S26A (*RPS26A*), and ribosomal protein L9A (*RPL9A*) in the target of rapamycin (TOR) signaling pathway. Gene expression levels of* RPS26A* and* RPL9A* in* uth1*, as well as in* uth1*,* sir2* double mutants, were significantly lower than those of the control group. Besides,* TORC1* gene expression in* uth1* mutant of K6001 yeast was inhibited significantly. These results suggested that parishin exhibited antiaging effects via regulation of Sir2/Uth1/TOR signaling pathway.

## 1. Introduction

The proportion of the world's population over 60 years old will be 22% in 2050 [1]. Aging-related diseases, such as Alzheimer's and Parkinson's diseases and diabetes, are becoming a severe threat to human health in aging society. Although many commercial available drugs are used to treat these diseases [2], they can only alleviate clinical symptoms and cannot cure the diseases. Therefore, a novel therapeutic strategy, such as antiaging, will be a promising technique to delay and prevent the occurrence of aging-related diseases.

Sir2 proteins are a family of proteins influencing the physiological responses and affecting the treatment of aging-related diseases. Increase of* SIR2* gene expression and activity can extend the life span of various model organisms [3–5]. More importantly, it can regulate oxidative stress by binding and deacetylation of FOXO transcription factors, which play a central role in regulating stress response [6]. Recently, sirtuin family has been considered a drug target for aging, metabolism, and aging-related diseases [7].

Most eukaryotes express two intracellular SODs, a Mn containing SOD2 in the mitochondrial matrix and a highly abundant Cu/Zn SOD1 that is largely cytosolic but is also found in the mitochondrial intermembrane space. SOD1 has function of protecting cells, regulating cell viability, and metabolism [8]. SOD2 takes an important role for antioxidative stress and scavenger of free radical.

**Figure 1:**
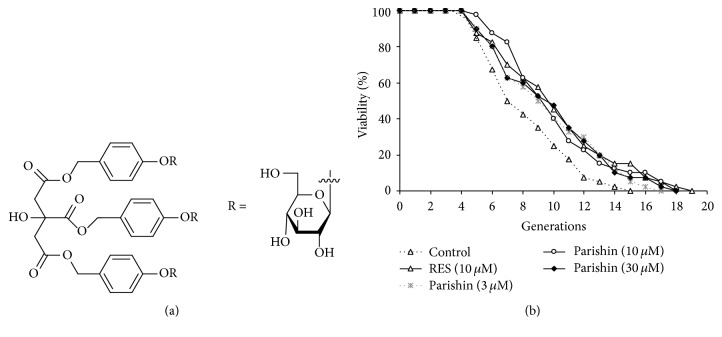
Chemical structure of parishin (a) and antiaging effects of parishin (b). For replicative lifespan assay, the yeast cells incubated in galactose medium were spread on glucose medium plates containing different concentrations of parishin. The daughter cells of 40 microcolonies in each plate were counted randomly. The assay was repeated at least thrice. The average lifespan of untreated K6001 was 7.38 ± 0.44 generations; resveratrol (RES) at 10 *μ*M, 9.23 ± 0.59^*∗*^; parishin at 3 *μ*M, 8.83 ± 0.56^*∗*^; parishin at 10 *μ*M, 9.20 ± 0.52^*∗∗*^; and parishin at 30 *μ*M, 8.98 ± 0.58^*∗*^. * ∗* and * ∗∗* indicate significant difference relative to the control (*p* < 0.05, *p* < 0.01).

*Gastrodia elata* (Tian Ma in Chinese) is an important traditional Chinese medicine. This herb has anticonvulsant, analgesia, calmness, hypnosis, nootropic, and anti-brain-aging functions for the central nervous system in traditional therapy of Chinese medicine [9]. In addition, it can promote the energy metabolism of myocardial cells;* G. elata* also has anti-inflammation effect and increases immunity [10, 11]. Many active ingredients, such as gastrodin, 4-hydroxybenzyl alcohol, 4-hydroxybenzaldehyde, benzyl alcohol, 4-hydroxy-3-methoxybenzaldehyde, 4-hydroxy-3-methoxybenzyl alcohol, parishin, and parishin B and parishin C, have been isolated from* G. elata* [12–16]. Among these compounds, gastrodin is a major active compound and has been developed to be a commercially available drug that is mainly used to treat neurasthenia-induced headache [17]. Parishin (Figure 1(a)), one of the major compositions of* G. elata*, can alleviate asthma [15]. However, its antiaging effects and mechanism of action have not been reported yet.

Yeast is a well-known bioassay model in antiaging research [18]. Recently, multiple conserved longevity pathways have been discovered in budding yeast [19]. Since it has the characters of short generation time, genetic tractability, and low costs, the budding yeast has become a premier model organism for aging research [20]. Replicative aging and chronological aging are used to assess longevity of yeast. The standard replicative lifespan assay needs to use micromanipulator to remove the daughter cells produced by one mother cell for every two hours. It is time consuming and labor intensive. Thus, it has become a rate-limited step on the progress of aging research. In 2004, Jarolim et al. established the replicative lifespan assay with K6001 yeast strain to improve the lifespan assay [18]. Recently, microfluidic technology for yeast replicative lifespan has also developed to address this problem [20].

In our previous studies, antiaging compounds, such as ganodermasides A–D, phloridzin, and nolinospiroside F, were isolated from* Ganoderma lucidum*, apple branches, and* Ophiopogon japonicus*, respectively, under a K6001 yeast bioassay system [21–24]. In the present study, parishin was isolated as a major antiaging composition from* G. elata* according to the same system. We report the isolation, structure elucidation, biological activity, and mechanism of action of parishin.

## 2. Materials and Methods

### 2.1. Isolation and Structure Elucidation of Parishin

The rhizomes of* G. elata* (dry weight: 200 g) were bought from Chengdu, Sichuan Province, China, and the identification of* G. elata* was confirmed. A voucher specimen (number 20110521) was kept at College of Pharmaceutical Sciences, Zhejiang University. They were ground and extracted with MeOH. The supernatant was separated via filtration and concentrated to obtain the methanol extract. The extract was partitioned between EtOAc and H_2_O. The H_2_O layer was concentrated to give 26 g of dried sample which was chromatographed on ODS (Cosmosil 75 C18-OPN, Nacalai Tesque, Ohtsu, Japan) and eluted with MeOH/H_2_O (20 : 80, 25 : 75, 30 : 70, 35 : 65, 40 : 60, 60 : 40, and 80 : 20) to afford 43 fractions. The active sample (1.4 g), which was eluted with MeOH/H_2_O (25 : 75, 30 : 70, and 35 : 65), was separated on silica gel (200–300 mesh, Yantai Chemical Industry Research Institute, Yantai, China) and eluted with CHCl_3_/MeOH (9 : 1, 8 : 2, 6 : 4, 5 : 5, 4 : 6, 3 : 7, 2 : 8, and 0 : 10) to afford 71 fractions. A portion (150 mg) of the active sample (921.0 mg), eluted with CHCl_3_/MeOH (6 : 4, 5 : 5, 4 : 6), was subjected to HPLC (Develosil ODS-UG-5 (*ϕ* 20/250 mm), Nomura Chemical, flow rate: 8 mL/min, MeOH/H_2_O (28 : 72)) to yield a pure active compound (100.0 mg, *t*_*R*_ = 36 min). The chemical structure of the compound was determined to be parishin by comparing ^1^H NMR, MS, and optical rotation data with those reported [14, 16]. ^1^H NMR (500 MHz, CD_3_OD): *δ* 2.78 (d, 2H, *J* = 15.0 Hz), 2.94 (d, 2H, *J* = 15.5 Hz), 3.40–3.52 (m, 12H), 3.70 (dd, 3H, *J* = 5.0, 12.0 Hz), 3.87 (dd, 3H, *J* = 1.5, 12.0 Hz), 4.90–5.01 (m, 9H), 7.04 (d, 2H, *J* = 8.5 Hz), 7.07 (dd, 4H, *J* = 1.5, 8.5 Hz), 7.16 (d, 2H, *J* = 8.5 Hz), 7.24 (dd, 4H, *J* = 2.5, 8.5 Hz); MS* m*/*z* 1019 (M + Na)^+^; [*α*]_*D*_^25^ = −73.0 (*c* 1.0, MeOH).

### 2.2. Yeast Strains, Media, and Lifespan Assay

The yeast strains used in present study were described in Table 1. The lifespan assay method and medium were similar to those previously reported [18]. To get enough yeast to do experiment, briefly, K6001 yeast strain was resuscitated in 5 mL of galactose medium and incubated in a shaking incubator at 160 rpm for 24–28 h at 28°C. About 1 mL of yeast culture was centrifuged for 3 min at 1,500 rpm. The yeast pellet was washed three times and diluted with phosphate buffer solution (PBS). After counting with a hemocytometer, approximately 4,000 cells were plated on glucose medium agar plates containing resveratrol (positive control, 10 *μ*M) or parishin (0, 3, 10, and 30 *μ*M). The plates were incubated at 28°C for 2 days, and 40 microcolonies formed on the plates were randomly observed under a microscope. The daughter cells produced by the mother cell were counted. The bioassay method of replicative lifespan of* sod1*,* sod2*,* uth1*, and* skn7* mutants with a K6001 background was identical to that of the K6001 strain.

### 2.3. Antioxidative Stress Assay

BY4741 yeast was treated with resveratrol (10 *μ*M) as a positive control or parishin (0, 3, 10, and 30 *μ*M) at 28°C for 48 h. Subsequently, about 0.1 OD of yeast cultures in each group was spotted on agar plates containing 9 mM of H_2_O_2_. The growth of yeast on the plate was observed and photographed after incubation at 28°C for 3 days.

To validate the accuracy of experiment, we used another method to examine the antioxidative stress of parishin again. BY4741 yeast was incubated for 24 h after it was treated with 0, 3, 10, and 30 *μ*M parishin or 10 *μ*M resveratrol; then it was treated with H_2_O_2_ at doses of 0 or 180 mM for 3 h. Approximately 0.1 OD of yeast in each group was washed with cold PBS buffer three times and treated in 15% ethanol for 20 min. The treated yeast cells were incubated with 10 *μ*g/mL propidium iodide at 37°C for 20 min in dark after washing with PBS buffer. Fluorescence microscope (Leica DMI 3000 B, Wetzlar, Germany) was used to observe the change of yeast cells under oxidative stress condition using an excitation wavelength of 535 nm and an emission wavelength of 615 nm. Approximately 100 cells were used to calculate the survival rate.

### 2.4. Determination of SOD Enzyme Activity, MDA, and ROS Level

BY4741 yeast cells were cultured in glucose medium after adding 0, 3, 10, or 30 *μ*M of parishin for 24 or 48 h. The SOD and MDA assays were performed as in the previous study [24]. After counting the yeast with a hemocytometer, approximately 1 × 10^9^ cells were washed thrice with PBS and resuspended in 1 mL of PBS. The cells were ultrasonicated (1 min for each time) for five times, followed by freeze and thaw (5 min in liquid nitrogen and subsequently 2 min in water bath at 37°C) and repeated sonication for five times. The cell lysates were centrifuged at 12,000 rpm at 4°C for 15 min, and the supernatant was removed to test the SOD activity and MDA level using SOD and MDA assay kits (Nanjing Jiancheng Bioengineering Institute, Nanjing, China), according to the manufacturer's instructions.

ROS assay was carried out using the method in the previous study [23]. BY4741 yeast cells were incubated in glucose medium with parishin (0, 3, 10, and 30 *μ*M) in a shaker incubator at 28°C for 23 h. Subsequently, DCFH-DA was added to 1 mL of the cells to get the final concentration of 40 *μ*M, and the cells were incubated in a shaker at 28°C in dark for 1 h. The cells were then washed thrice with PBS quickly, and the DCF fluorescence magnitude of 1 × 10^7^ cells was detected by a fluorescent plate reader using excitation and emission wavelengths of 488 and 525 nm, respectively.

### 2.5. RT-PCR Analysis

BY4741,* uth1*, and* uth1*,* sir2* double mutants with a BY4741 background were treated with control or different concentrations of parishin and cultured in glucose medium in an incubator at 28°C overnight with shaking. Wild type and* uth1* mutant of K6001 were cultured in galactose medium in a shaker at 28°C overnight. Cells were collected, and RNA was extracted via the hot-phenol method. RNA was purified with a RNApure tissue kit (Beijing Cowin Biotech Company, Beijing, China), and reverse transcription was performed using a HiFi-MMLV cDNA kit (Beijing Cowin Biotech Company, Beijing, China) with 5 *μ*g of total RNA. RT-PCR was conducted similarly to that of the previous study [23]. CFX96-Touch (Bio-rad, Hercules, USA) and SYBR Premix EX Taq (Takara, Otsu, Japan) were used. The thermal cycling parameters for* RPS26A* and* RPL9A* are as follows: 40 cycles, 95°C for 15 s and 60°C for 35 s; for* SIR2*, the parameters are as follows: 40 cycles, 94°C for 15 s, 60°C for 25 s, and 72°C for 20 s; for* TORC1*, the parameters are as follows: 40 cycles, 95°C for 15 s, 59°C for 25 s, and 72°C for 20 s. The primers used for RT-PCR are as follows: for* RPS26A*, sense 5′-TCA GAA ACA TTG TTG AAG CCG C-3′ and antisense 5′-ACA ATT CTG GCG TGA ATA GCA C-3′; for* RPL9A*, sense 5′-ATG GTG CCA AAT TCA TTG AAG TC-3′ and antisense 5′-AGT TAC CTG ACA AGA CAA TTT CG-3′; for* SIR2*, sense 5′-CGT TCC CCA AGT CCT GAT TA-3′ and antisense 5′-CCA CAT TTT TGG GCT ACC AT-3′; for* TORC1*, sense 5′-TTG GTA CAA GGC ATG GCA TA-3′ and antisense 5′-TAC CGT CAA TCC GCA CAT TA-3′; and for* TUB1*, sense 5′-CCA AGG GCT ATT TAC GTG GA-3′ and antisense 5′-GGT GTA ATG GCC TCT TGC AT-3′. Relative gene expression data were analyzed using 2^−ΔΔCt^ method. The amounts of* RPS26A*,* RPL9A*,* TORC1*, and* SIR2* mRNA were normalized to that of* TUB1*.

### 2.6. Statistical Analysis

Significant differences among groups in all experiments were determined by analysis of variance, followed by two-tailed multiple *t*-tests with Bonferroni correction using SPSS biostatistics software. A *p* value of less than 0.05 was considered statistically significant.

## 3. Results and Discussion

### 3.1. Parishin Extends the Replicative Lifespan of Yeast

K6001, a mutant strain of yeast with W303 background expresses* CDC6*, an essential gene for growth, under control of the mother-specific* HO* promoter and a galactose-dependent promoter* GAL1-10*. When K6001 cells are cultured in galactose,* GAL1-10*::*CDC6* is expressed both in mother and in daughter cells; however, when the expression of* GAL1-10*::*CDC6* gene is repressed by glucose, only the mother cell-specific expression of* HO*::*CDC6* remains to support growth [18]. Due to its specificity above, the replicative lifespan assay of K6001 is much more efficient and easier to manipulate than that of the other yeasts. It has been used to evaluate antiaging activity of compounds. By employing this bioassay system, several antiaging substances such as ganodermasides A–D, phloridzin, and nolinospiroside F were isolated. In the present study, this bioassay system was used to guide the isolation of an antiaging substance from* G. elata*. The changes on the replicative lifespan of K6001 yeast after parishin treatment at various doses are displayed in Figure 1(b). Parishin significantly extended the replicative lifespan of K6001 at 3, 10, and 30 *μ*M (*p* < 0.05, *p* < 0.01, and *p* < 0.05, resp.). These results suggested that parishin had antiaging effects. In addition, we also performed kinetics of growth assay of yeast under the influence of parishin. Significant changes were not observed in resveratrol treatment group and parishin treatment group (see Supplementary Figure  1 in Supplementary Material available online at http://dx.doi.org/10.1155/2016/4074690). At this point, it is possible that the kinetics of growth assay is not suitable to assess the replicative lifespan of yeast.

### 3.2. Parishin Enhances Gene Expression of* SIR2*

**Figure 2:**
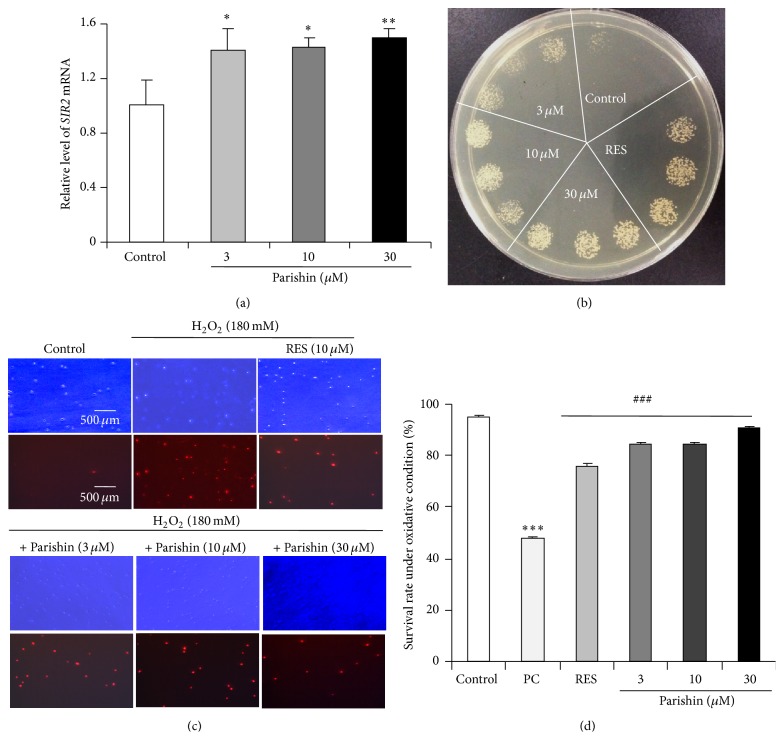
Effects of parishin on the gene expression of* SIR2* (a) in normal condition and growth of BY4741 yeast under oxidative stress conditions (b, c, and d). The change on* SIR2* gene expression in yeast after treatment with parishin at doses of 0, 3, 10, and 30 *μ*M (a). Amount of* SIR2* mRNA was normalized to that of* TUB1*. The effects of parishin on yeast growth under oxidative stress induced by H_2_O_2_ (b). BY4741 yeast was incubated for 48 h after it was treated with 0, 3, 10, and 30 *μ*M parishin or 10 *μ*M resveratrol; then about 5 *μ*L of the same concentration of yeast was dropped onto glucose medium agar plates containing 9 mM H_2_O_2_. The yeast was incubated for 3 d at 28°C and photographed. The micrographs (c) and survival rate (d) of yeast under oxidative stress condition. BY4741 yeast was incubated for 24 h after it was treated with 0, 3, 10, and 30 *μ*M parishin or 10 *μ*M resveratrol; then it was treated by H_2_O_2_ at doses of 0 or 180 mM for 3 h. Approximately 0.1 OD of yeast in each group was washed with cold PBS for three times and treated in 15% ethanol for 20 min. The treated yeast cells were incubated with propidium iodide at 10 *μ*g/mL for 20 min after washing with PBS. Fluorescence microscope was used to observe the change of yeast cells under oxidative stress condition using an excitation wavelength of 535 nm and an emission wavelength of 615 nm. Approximately 100 cells were used to calculate the survival rate. Each experiment was performed at least three times. PC represents positive control treated with 180 mM H_2_O_2_ for 3 h. *∗*, *∗∗*, and *∗∗∗* indicate significant difference relative to the corresponding control (*p* < 0.05, *p* < 0.01, and *p* < 0.001, resp.). * *### represents significant difference relative to the positive control (*p* < 0.001).

*SIR2* gene is one of the most important longevity genes. The increase of* SIR2* gene expression or enzyme activity could extend the yeast lifespan [3]. Therefore, we examined the gene expression of* SIR2* in yeast treated with parishin. As expected, the gene expression levels of* SIR2* in parishin treatment groups were significantly increased (Figure 2(a); *p* < 0.05, *p* < 0.05, and *p* < 0.01). This result suggested that* SIR2* gene was involved in the antiaging effects of parishin.

### 3.3. Parishin Improves the Survival Rate of Yeast under Oxidative Stress Conditions

Oxidative stress is one of the most important factors for aging, and oxidative free radicals do harm to cellular constituents, such as DNA, proteins, carbohydrates, and lipids [25]. Therefore, we focused on this point to measure the parameters related to antioxidation in yeast. As shown in Figure 2(b), parishin significantly increased the number of colonies of yeast. Moreover, the viability of yeast after treatment with parishin at doses of 3, 10, and 30 *μ*M was notably increased compared with positive control group under oxidative stress condition (Figures 2(c) and 2(d); *p* < 0.001, *p* < 0.001 and *p* < 0.001, resp.). We also used agar plates to examine the survival rate of yeast under oxidative stress, and the same results were obtained (Supplementary Figure  2). These results suggested that antioxidation played an important role in the antiaging effect of parishin.

### 3.4. Parishin Increases SOD Enzyme Activity of Yeast and Decreases ROS and MDA Levels

**Figure 3:**
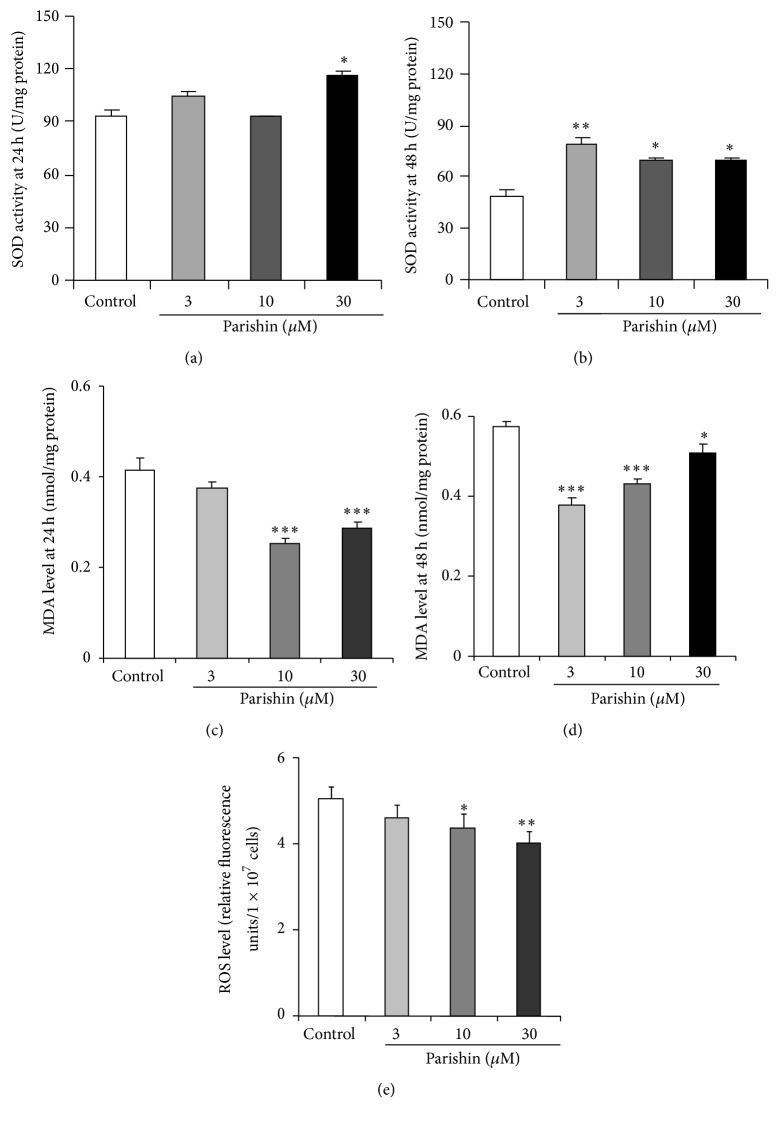
Effects of parishin on SOD enzyme activity (a, b), MDA (c, d), and ROS levels (e). The change on SOD enzyme activity after treating parishin at various doses for 24 h (a) or 48 h (b), respectively. BY4741 yeast cells were disintegrated by ultrasonication and freeze-thawing for five times, followed by repeated ultrasonication for five times. Cell lysate was centrifuged and the supernatant was removed for measurement of SOD activity using a SOD assay kit. Effect of parishin on MDA in yeast after parishin treatment at various doses for 24 h (c) or 48 h (d). BY4741 yeast cells were cultured for 24 h or 48 h and disintegrated as described in SOD assay, and changes in MDA level were measured with an MDA assay kit. Effect of parishin on ROS level of yeast (e). BY4741 yeast cells were incubated with parishin for 23 h. Subsequently, DCFH-DA was added into culture medium to a final concentration of 40 *μ*M and incubated for 1 h. The intensity of DCF of yeast was detected with a fluorescence plate reader. *∗*, *∗∗*, and *∗∗∗* indicate significant difference from corresponding control (*p* < 0.05, *p* < 0.01, and *p* < 0.001).

SOD is an important enzyme that participates in free radical scavenging. Thus, we measured the SOD activity in yeast after parishin treatment. The SOD enzyme activity of yeast was only significantly increased in yeast after treatment with 30 *μ*M of parishin for 24 h (Figure 3(a); *p* < 0.05). However, the significant increases of SOD activity in yeast were observed after administrating parishin at 3, 10, and 30 *μ*M for 48 h (Figure 3(b); *p* < 0.01, *p* < 0.05, and *p* < 0.05, resp.).

ROS are byproducts of oxidative metabolism and important cause of aging [25]. Hence, we tested the change on ROS accumulation in yeast after parishin treatment. The ROS levels in yeast were decreased significantly at 10 and 30 *μ*M of parishin treatment (Figure 3(e); *p* < 0.05 and *p* < 0.01, resp.).

MDA, as the main degradation product of polyunsaturated lipids [26], causes considerable harm to organisms. It can damage membrane, add fluidity to cells, and also influence the DNA [26–28]. Therefore, we investigated the change on MDA level in yeast after treatment with parishin at 24 and 48 h. The MDA levels of yeast at 24 h in Figure 3(c) were significantly decreased after treatment with parishin at doses of 10 and 30 *μ*M (*p* < 0.001, *p* < 0.001), compared with control group. The significant reduction of MDA levels in 3, 10, and 30 *μ*M parishin-treated groups were observed at 48 h (Figure 3(d); *p* < 0.001, *p* < 0.001, and *p* < 0.05). These results again suggested that antioxidative stress had an important role in the antiaging activity of parishin.

### 3.5. Parishin Does Not Affect the Lifespans of* Sod1*,* Sod2*,* Uth1*, and* Skn7* Mutants with a K6001 Background

**Figure 4:**
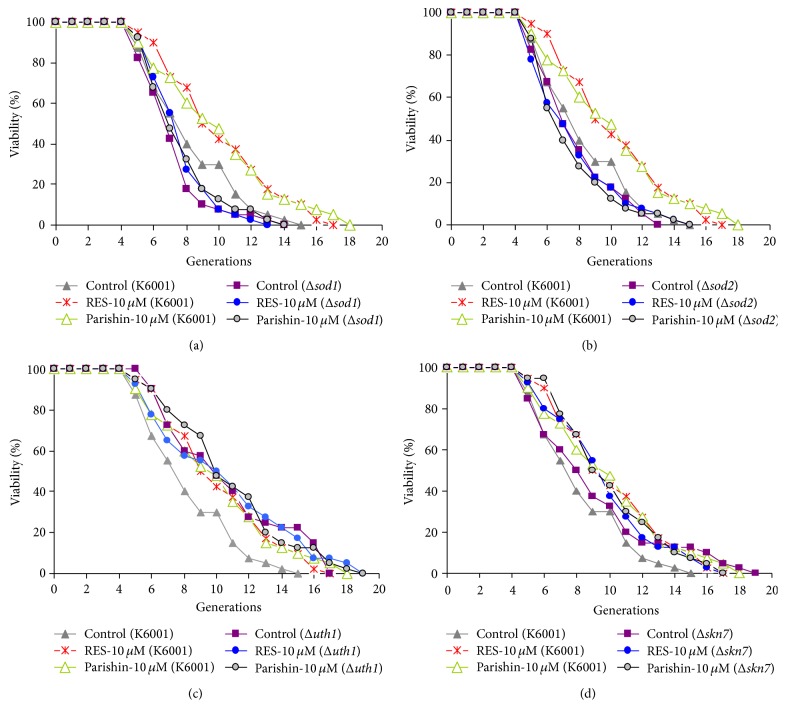
Effects of parishin on the replicative lifespan of* sod1* (a),* sod2* (b),* uth1* (c), and* skn7* (d) mutant yeast strains with a K6001 background. The daughter cells of 40 microcolonies of each experiment were counted. The assay was repeated at least thrice. The results were displayed as mean ± SEM. The average lifespan of untreated K6001 was 7.25 ± 0.26 generations; RES at 10 *μ*M, 9.25 ± 0.29^*∗∗*^; and parishin at 10 *μ*M, 9.13 ± 0.30^*∗∗*^. (a) Δ*sod1* was 6.35 ± 0.22; RES at 10 *μ*M, 6.80 ± 0.22 and; parishin at 10 *μ*M, 6.83 ± 0.23. (b) Δ*sod2* was 6.90 ± 0.24; RES at 10 *μ*M, 6.80 ± 0.26; and parishin at 10 *μ*M, 6.60 ± 0.24. (c) Δ*uth1* was 9.73 ± 0.31; RES at 10 *μ*M, 9.58 ± 0.32; and parishin at 10 *μ*M, 9.93 ± 0.30. (d) Δ*skn7* was 8.20 ± 0.31; RES at 10 *μ*M, 8.83 ± 0.28; and parishin at 10 *μ*M, 9.05 ± 0.29. Δ*sod1*, Δ*sod2*, Δ*uth1*, and Δ*skn7* represent* sod1*,* sod2*,* uth1,* and* skn7* mutant yeast strain with a K6001 background, respectively. *∗∗* indicates significant difference compared with untreated K6001 (*p* < 0.01).

*SOD* gene is an important antioxidative stress gene that participates in free radical scavenging. To confirm whether* SOD* gene participated in the antiaging effect of parishin, we used* sod1* and* sod2* mutants with a K6001 background to examine the effects of parishin on replicative lifespan. The lifespan of s*od1* and* sod2* mutants was shorter than that of K6001 yeast strains, and parishin did not affect the replicative lifespan of these mutants (Figures 4(a) and 4(b)). These results indicated that* SOD* gene played an important role in the antiaging effect of parishin.

*UTH1* was an aging gene related to oxidative stress, and* UTH1* inactivation increased resistance to oxidants [29]. In addition,* SKN7* is the transcriptional activator of* UTH1.* Thus, we used* uth1* and* skn7* mutants with a K6001 background to investigate whether these two genes were involved in the lifespan extension of parishin. The longer replicative lifespan of* uth1* mutant of K6001 yeast was observed in our present study as another report [30]. After administrating parishin, the changes on replicative lifespan of these mutants were not observed (Figures 4(c) and 4(d)). These results revealed that* UTH1* and* SKN7* genes were involved in the antiaging effect of parishin.

### 3.6. Parishin Inhibits the* TORC1*,* RPS26A*, and* RPL9A* Gene Expressions, Downstream of the TOR Signaling Pathway

**Figure 5:**
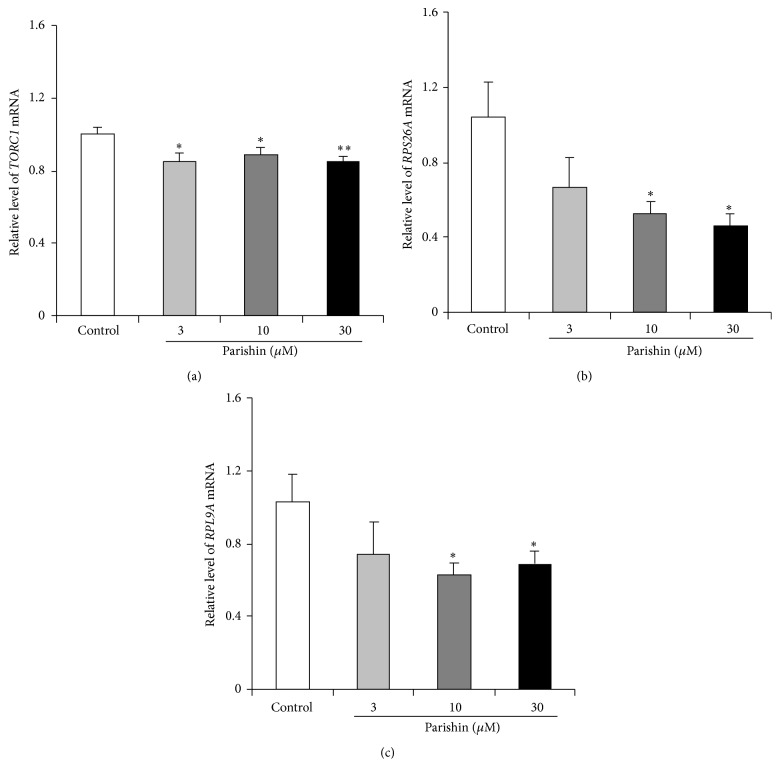
Effects of parishin on target of rapamycin (TOR) signaling pathway. Parishin significantly reduced the gene expressions of* TORC1* (a),* RPS26A* (b), and* RPL9A* (c). Amounts of the mRNA above were normalized to that of* TUB1*. The results were displayed as mean ± SEM for three independent experiments. *∗* and *∗∗* indicate significant difference between the control and treatment groups (*p* < 0.05, *p* < 0.01).

TOR signaling pathway has prominent importance in regulating the process of aging. This signaling pathway controls growth-related processes, including regulation of translation, ribosome biogenesis, amino acid permease stability, and induction of autophagy [31]. TOR inhibition leads to the decreasing expression of some ribosomal protein genes, such as* RPS26A* and* RPL9A*, and increasing expression of some genes coding permeases for nitrogenous compounds, such as* GAP1* and* MEP2* [32]. TOR also regulates ribosome maturation via the nuclear GTP-binding protein NOG1 [33]. Therefore, we detected the effects of parishin on TOR signaling pathway using RT-PCR analysis. The changes on* TORC1*,* RPS26A*, and* RPL9A* gene expressions were presented in Figures 5(a)–5(c).* TORC1* gene expression was significantly decreased after parishin treatment at doses of 3, 10, and 30 *μ*M (Figure 5(a); *p* < 0.05, *p* < 0.05, and *p* < 0.01, resp.), and the gene expressions of* RPS26A* and* RPL9A* were also inhibited by parishin at doses of 10 and 30 *μ*M (Figures 5(b) and 5(c); *p* < 0.05 and *p* < 0.05). These results revealed that TOR signaling pathway may be involved in the antiaging effects of parishin. However, significant difference was not observed in* NOG1*,* GAP1*, and* MEP2* gene expressions in yeast after parishin treatment (data not shown). Possibly, parishin did not block the TOR signaling pathway completely.

### 3.7. *UTH1* Gene Regulates TOR Signaling Pathway

**Figure 6:**
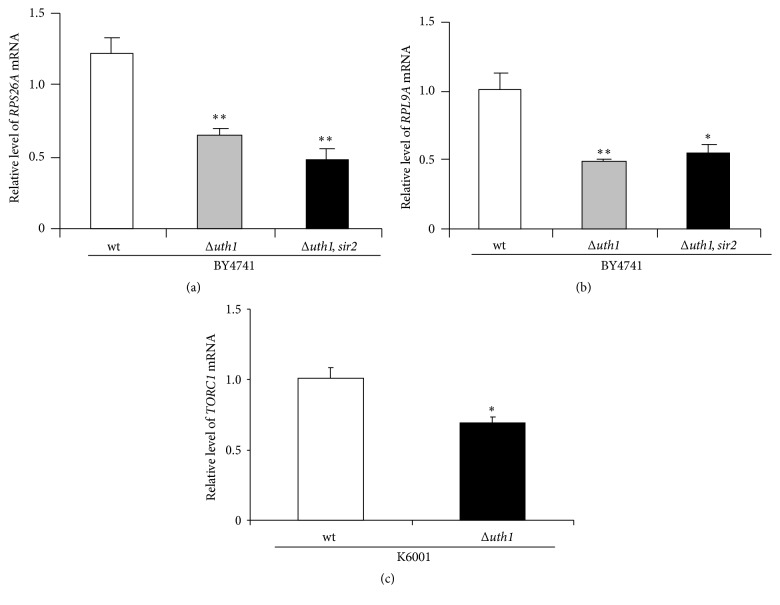
Interaction between* UTH1* gene and TOR signaling pathway. The change on* RPS26A* (a) and* RPL9A* (b) gene expressions in wild type,* uth1* mutant, and* uth1*,* sir2* double mutant with BY4741 background. The change on* TORC1* gene expressions in wild type and* uth1* mutants with K6001 background (c). Amounts of the mRNA above were normalized to that of* TUB1*. Each experiment was carried out in triplicate independently. *∗* and *∗∗* indicate the significant difference between the control and treatment groups (*p* < 0.05, *p* < 0.01).

In the present study, both Uth1 and TOR signaling pathways participated in the antiaging effects of parishin. To investigate whether they had interactions, we investigated the gene expression of* RPS26A* and* RPL9A* in* uth1* as well as in* uth1*,* sir2* double mutant with BY4741 background. As expected, their gene expression in these mutants was significantly decreased comparing with control group, respectively (Figures 6(a) and 6(b); *p* < 0.01, *p* < 0.01, and *p* < 0.01, *p* < 0.05, resp.). Furthermore, we detected the gene expression of* TORC1* in* uth1* mutants with K6001 background. Significant reduction of* TORC1* gene expression was observed in the* uth1* mutant with K6001 background (Figure 6(c); *p* < 0.05). In addition, another research also indicated that* UTH1* could affect TOR signaling pathway [34]. These results clarified that Uth1 could regulate TOR signaling pathway. Similarly, we did not find significant changes on* NOG1*,* GAP1*, and* MEP2* gene expression in* uth1* mutant as well as* uth1*,* sir2* double mutants of BY4741 (data not shown).

## 4. Conclusion

In summary, we isolated parishin, an antiaging compound from* G. elata*, using the replicative lifespan assay of K6001. Parishin could significantly extend the lifespan of yeast via antioxidative stress, increase the* SIR2* gene expression, and inhibit the Uth1/TOR signaling pathway. Furthermore, Uth1 can upregulate TOR signaling pathway. Thus, parishin might be a valuable lead compound for drug discovery against age-related diseases.

## Supplementary Material

In the supplementary material, the methods and the results of kinetics of K6001 yeast growth and another antioxidative stress assay were presented.

## Figures and Tables

**Table 1:** Strains used in this study.

Strains	Genotype	Source or reference
K6001	*MATa*, *ade2-1*, *trp1-1*, *can1-100*, *leu2-3*, *112*, *his3-11*, *15*, *GAL*, *psi*+, *ho::*HO*::CDC6* (at HO), *cdc6::hisG*, *ura3::URA3 GAL-ubiR-CDC6* (at *URA3*)	[18, 35]

Δ*uth1* of K6001	Replace the *UTH1* gene in K6001 with kanamycin gene	Constructed by Professor Akira Matsuura

Δ*skn7* of K6001	Replace the *SKN7* gene in K6001 with kanamycin gene	Constructed by Professor Akira Matsuura

Δ*sod1* of K6001	Replace the *SOD1* gene in K6001 with kanamycin gene	Constructed by Professor Akira Matsuura

Δ*sod2* of K6001	Replace the *SOD2* gene in K6001 with kanamycin gene	Constructed by Professor Akira Matsuura

BY4741	*MATa*, *his3*Δ*1*, *leu2*Δ*0*, *met15*Δ*0*, *ura3*Δ*0*	[36]

Δ*uth1* of BY4741	Replace the *UTH1* gene in BY4741 with kanamycin gene	Constructed by Professor Akira Matsuura

Δ*uth1*, *sir2* of BY4741	Replace the *UTH1* gene and *SIR2* gene in BY4741 with kanamycin gene	Constructed by Professor Akira Matsuura
